# CEA-targeted CAR-NK cells derived from embryonic stem cells exhibit potent anti-tumor activity against colorectal cancer

**DOI:** 10.3389/fimmu.2026.1901058

**Published:** 2026-08-28

**Authors:** Gaohua Li, Lingping Zhao, Jingzhi Shen, Haiyang Wang, Jinru He, Yiming Liu, Ruge Zang, Tiantian Cui, Quan Zeng, Junnian Zhou, Wen Yue, Yanan Wang, Jiafei Xi

**Affiliations:** 1 Department of Retroperitoneal Tumor Surgery, Peking University People’s Hospital, Beijing, China; 2 Academy of Military Medical Sciences, Beijing, China; 3 Guangdong Provincial Key Laboratory of Precision Medicine for Gastrointestinal Tumor, Nanfang Hospital, Southern Medical University, Guangzhou, China

**Keywords:** carcinoembryonic antigen, CAR-NK cells, colorectal cancer, embryonic stem cells, immunotherapy

## Abstract

**Background:**

Carcinoembryonic antigen (CEA) is overexpressed in most colorectal cancers (CRC) and serves as a promising immunotherapeutic target. However, current immune cell therapies against CRC show limited clinical efficacy, and the lack of renewable, standardized cell sources hampers broader application. The present study aimed to establish a scalable source of CEA-targeted natural killer (NK) cells from engineered human embryonic stem cells (hESCs).

**Methods:**

hESCs were genetically engineered to express a CEA‑specific chimeric antigen receptor (CAR). The modified hESCs were subsequently differentiated into NK cells, yielding a homogeneous population of CAR hESC-NK cells. *In vitro* cytotoxicity against CEA-high CRC cell lines was evaluated using LDH and IFN-γ release assays. For *in vivo* assessment, we established a xenograft mouse model bearing CEA-positive CRC tumors. Following adoptive transfer of CAR-hESC-NK cells, tumor growth was monitored by bioluminescence imaging, overall survival was recorded for Kaplan–Meier analysis, and the persistence of transferred cells in peripheral blood was examined by flow cytometric analysis at serial time points.

**Results:**

*In vitro*, CAR hESC‑NK cells demonstrated potent and specific cytotoxicity against CEA-high CRC cell lines, along with significantly enhanced cytokine secretion compared to unmodified NK cells. In the xenograft model, adoptive transfer of CAR hESC‑NK cells resulted in marked tumor growth inhibition and prolonged survival, with no observable off‑target toxicity. Importantly, the CAR CEA‑ESC line maintained stable transgene expression and could serve as a renewable seed cell source.

**Conclusion:**

We have established a scalable platform for producing CEA‑targeted NK cells from engineered hESCs, providing an off‑the‑shelf, homogeneous cell product with robust antitumor activity. This strategy provides effective and specific immunotherapy against CEA-positive colorectal cancer and holds potential for other CEA-expressing malignancies.

## 1 Introduction

Colorectal cancer (CRC) is one of the most common malignant tumors worldwide, with persistently high morbidity and mortality rates (1, 2). Despite continuous updates and advancements in traditional treatment modalities such as surgery, radiotherapy, and chemotherapy (3), the five-year survival rate for patients with colorectal cancer remains unsatisfactory at approximately 15% (4). Although new chemotherapeutic and targeted drugs have been introduced in recent years, the population of patients who benefit from these breakthrough agents remains limited (5, 6). A limited proportion of CRC patients exhibiting mismatch repair-deficient or microsatellite instability-high molecular subtypes are likely to benefit from emerging immune checkpoint inhibitor therapies (7, 8). The scientific community is actively investigating the mechanisms underlying resistance to immunotherapy and seeking to expand its applicability in CRC. Therefore, there is an urgent need to explore novel and effective therapeutic strategies to address clinical demands.

The development of cell-based immunotherapies represents a promising avenue worthy of further investigation. Adoptive cell therapy has revolutionized the treatment of hematologic malignancies, with chimeric antigen receptor (CAR) T cells achieving remarkable efficacy in relapsed/refractory B-cell leukemias and lymphomas (9, 10). However, CAR T cells have shown limited efficacy in solid tumors, including CRC, due to several barriers such as poor trafficking into the immunosuppressive tumor microenvironment (TME), and significant adverse events including cytokine release syndrome and neurotoxicity (11). In contrast, CAR-natural killer (NK) cells therapy has emerged as a cutting-edge option for cellular immunotherapy of CRC due to its inherent multimodal killing mechanism and advantages in overcoming immunosuppression (12–15). In primary and metastatic CRC tissues, carcinoembryonic antigen (CEA) is predominantly localized on the apical membrane of malignant colonic epithelial cells, with high-level expression observed in over 90% of CRC cases (16, 17). NK cells do not inherently recognize CEA but rather make functional decisions by integrating a series of activation and inhibition signals. Down-regulation of MHC class I molecule expression on the surface of CRC cells, together with increased expression of NKG2D ligands, jointly enables NK cell recognition and killing of CRC cells (18, 19). Preclinical studies have demonstrated that CEA-targeting CAR constructs can effectively enhance the killing ability of NK cells against CRC cells. For example, CEA-CAR NK cells efficiently infiltrate CRC tumor sites, persist for extended durations when engineered with membrane-bound IL-15, and synergize with immune checkpoint blockade to overcome immunosuppressive TME barriers (20–22).

NK cells offer the advantage of serving as allogeneic off-the-shelf products, thereby obviating the need for patient-specific preparation (23, 24). In current research, sources of NK cells include primary NK cells from peripheral blood (PB-NK), NK92 cell lines, and NK cells from umbilical cord blood (UCB). However, PB-NK cells present challenges related to the high cost of large-scale collection and GMP-grade separation processes, as well as unstable sources from different donors (25). The NK92 cell line is of tumor origin and requires irradiation treatment before injection to eliminate the impact of its proliferative capacity on sustained anti-tumor effects (26). UCB-NK cells require complex induction processes and exhibit inherent functional limitations (27, 28). Using human pluripotent stem cells as seed cells to generate NK cells can further realize the advantage of “off-the-shelf” availability (24). Two types of human pluripotent stem cells currently under exploration for clinical use are human embryonic stem cells (hESCs) and human induced pluripotent stem cells (29). Utilizing hESCs to obtain NK cells represents an emerging cutting-edge technical approach in recent years. Its greatest advantage lies in the ability to perform CAR modification at the initial seed cell stage, subsequently inducing differentiation to obtain large numbers of uniformly characterized CAR-NK cells for tumor immunotherapy, which holds broad application prospects (30).

Therefore, in this study, we constructed a CEA-specific CAR and transfected it into hESCs, which were then induced to differentiate into CAR hESC-NK cells. These cells demonstrated significant anti-tumor activity and favorable safety against colorectal cancer, providing effective preclinical evidence for CAR hESC-NK cell therapy in CRC treatment.

## 2 Materials and methods

### 2.1 Experimental cells and culture

Human 293T (Cat. No. CL-0005), Caco-2 (Cat. No. CL-0050), SW1116 (Cat. No. CL-0307), SW837 (Cat. No. CL-0485B), and NK92 (Cat. No. CL-0530) cell lines were purchased from Wuhan Procell Biotechnology Co., Ltd. (Wuhan, Hubei, China). 293T and Caco-2 cells were cultured in DMEM (D6429, Sigma-Aldrich) supplemented with 10%FBS (16000-044, Gibco). SW1116 and SW837 cells were maintained in Leibovitz’s L-15 medium (11415064, Gibco) supplemented with 10% FBS. NK92 cells were cultured in α-MEM (M4526, Sigma-Aldrich) prepared as follows (per 100 mL): 2mM glutamine (G103979, aladdin), 0.15 g NaHCO_3_ (S432108, aladdin), 0.2mM inositol (I656931, aladdin), 0.1mM β-mercaptoethanol (M3148, Sigma-Aldrich), 0.02mM folic acid (S4605, Selleck), 200 U/mL recombinant IL-2 (130-097-743, Miltenyi Biotec), 20mL horse serum (26050070, Gibco), and 20mL FBS. hESCs were maintained in DMEM/F12 medium (C11330500BT, Gibco) (prepared as a 200 mL system) supplemented with 40mL KSR (10828028, Gibco), 2mL NEAA (M7145, Sigma-Aldrich), 2mM glutamine, 0.1mM β-mercaptoethanol, 10ng/mL bFGF (130-093-838, Miltenyi Biotec), and 2mL PS (15140122, Gibco).

### 2.2 CAR-CEA vector construction

CEA was selected as the target antigen. The single-chain variable fragment (scFv) was derived from the anti-CEA monoclonal antibody MEF23 (ab197070, Abcam, USA) via receptor–ligand interaction-based synthesis. The CAR-CEA construct was engineered to sequentially encode a signal peptide (SP), the anti-CEA scFv, a CD8α hinge region, a CD28 transmembrane domain, a CD28 intracellular co-stimulatory domain, and a CD3ζ signaling domain. The complete CAR cassette was cloned into the pCDH-CMV-MCS-EF1α-GFP lentiviral vector using the XbaI and BamHI restriction sites. The viral expression vector was synthesized by Nanjing Jingbai Biotechnology Co., Ltd.

### 2.3 Generation of the CAR-CEA hESC cells line

Prior to transfection, hESCs were cultured at a density of 2 × 10^5^ cells/mL and passaged every 2–3 days. Viral supernatant was added to the hESC cultures, followed by the addition of polybrene. The mixture was gently agitated and incubated at 37 °C in a 5% CO_2_ atmosphere. GFP expression was detectable after approximately 12 hours. After 24 hours, the medium was replaced with fresh hESC culture medium. At 48 hours post-transduction, cells were passaged and expanded, and GFP-positive cells were isolated via FACS to obtain CAR-CEA hESCs. This initial sorting step serves to enrich for CAR-positive hESCs from the bulk transduced cell population. Following an additional 1–2 weeks of culture, a second round of FACS was performed to enrich for CAR-CEA hESC cells stably expressing GFP. This second sorting yields a clonally homogeneous population with uniform CAR/GFP expression, ensuring consistent NK differentiation efficiency.

### 2.4 Induction method of CAR-CEA hESC into NK cells

The derivation of NK cells from ESCs and CAR transfected ESCs comprised three phases over 30 days. All induction steps were based on the protocol established by (31). Briefly, ESCs were seeded in 96-well round-bottom plates (plate 650101, lid 656161, greiner bio-one) at a density of 4,000 cells per well in 100 µL of BPEL medium containing 40 ng/ml SCF (130-096-692, Miltenyi biotec), 20 ng/ml VEGF (130-093-825, Miltenyi biotec), and 20 ng/ml BMP-4 (130-111-168, Miltenyi biotec). After day 6 of hematopoietic differentiation, spin embryoid bodies (EBs) were then directly transferred into gelatin-coated 24-well plates, achieving a density of 8–12 EBs per well. The final volume in each well was adjusted to approximately 1 mL with BEL medium containing 50ng/mL SCF, 40 ng/mL VEGF, 30 ng/mL IL-3 (130-093-909, Miltenyi biotec), 30 ng/mL IL-6 (130-093-929, Miltenyi biotec), 30 ng/mL TPO (130-095-745, Miltenyi biotec), and 30 ng/mL EPO (130-095-745, Miltenyi biotec) for 8 days. OP9-DL1 (YS1763C) cell lines were purchased from Shanghai Yaji Biotechnology Co., Ltd. Subsequently, cells harvested from the 24-well were seeded at a density of 2×10^6^ cells/mL onto the pre-plated OP9-DL1 feeder layer in 6-well plates. Half of the medium was refreshed twice per week, and fresh OP9-DL1 stromal cells were replenished weekly. Cells were then further differentiated into NK cells using 5 ng/mL IL-3 (first week only), 10 ng/mL IL-15 (130-095-760, Miltenyi biotec), 20 ng/mL IL-7 (130-095-367, Miltenyi biotec), 20 ng/mL SCF, and 10 ng/mL Flt3L (130-096-474, Miltenyi biotec) for 21 days.

### 2.5 Induction method of umbilical cord blood mononuclear cells to NK cells

The UCB used in this study was obtained from the Beijing Umbilical Cord Blood Bank. All procedures involving human subjects in this study were approved by the Ethics Committee of the Academy of Military Medical Sciences (AMMS; Approval No. AF/SC-08/02.160). Fresh blood was mixed with one-third volume of erythrocyte sedimentation solution and allowed to stand for 30 min. The supernatant was then layered onto lymphocyte separation medium and centrifuged at 2,000 rpm for 20 min to obtain mononuclear cells. Cells were seeded into low-attachment 6-well plates at 2 × 10^6^ cells/mL in NK induction medium and left undisturbed for the first 3 days. From day 4 onward, half-medium changes were performed every other day until day 14 to yield mature NK cells.

### 2.6 LDH cytotoxicity assay

Cytotoxicity was assessed using a lactate dehydrogenase (LDH) release assay according to the manufacturer’s protocol (CytoTox 96^®^ Non-Radioactive Cytotoxicity Assay, Promega). Briefly, target cells were seeded in 96-well plates at an optimized density and co-cultured with effector cells at indicated effector-to-target (E:T) ratios. After incubation for 4–6 h at 37 °C, plates were centrifuged at 250g for 5 min, and 50 µL of supernatant from each well was transferred to a fresh 96-well flat-bottom plate. An equal volume of LDH substrate mix was added to each well and incubated for 30 min at room temperature in the dark. Absorbance was measured at 490 nm using a microplate reader. Spontaneous LDH release (target cells alone) and maximum LDH release (target cells lysed with 1% Triton X-100) were included as controls. All assays were performed in triplicate and repeated in at least three independent experiments.

### 2.7 IFN-γ release assay

NK cell effector function was assessed by measuring interferon-gamma (IFN-γ) secretion. Briefly, NK cells (effectors) were co-cultured with target cells at an effector-to-target (E:T) ratio of 5:1 in 96-well U-bottom plates using RPMI-1640 medium supplemented with 10% FBS and 50 IU/mL recombinant human IL-2. For positive control stimulation, NK cells were treated with 25 ng/mL phorbol 12-myristate 13-acetate (PMA) (Selleck) and 1 µg/mL ionomycin (S1672, Beyotime). After 4–6 h of incubation at 37 °C in 5% CO_2_,1 µg/mL brefeldin A (S1536, Beyotime) or 2 µM monensin (S1753, Beyotime) was added for intracellular protein retention, followed by an additional 12-16h of culture. Alternatively, cell-free supernatants were harvested after 24 h of co-culture without retention agents for extracellular detection. IFN-γ concentrations in supernatants were quantified using a human IFN-γ ELISA kit (P1521, Beyotime) according to the manufacturer’s instructions. Absorbance was measured at 450 nm, and cytokine concentrations were interpolated from a standard curve. All experiments were performed in triplicate and repeated independently at least three times.

### 2.8 Animal preparation

Male SPF-grade NOD SCID mice (8–12 weeks old) were obtained from Sibeifu Biotechnology Co., Ltd. (Beijing, China). Animals were housed under a 12 h light/dark cycle with ad libitum access to food and water and were allowed to acclimatize for one week prior to experimental procedures. All animal experiments were approved by the Animal Care and Use Committee of the Academy of Military Medical Sciences (Approval No. IACUC-DWZX-2022-557) and conducted in accordance with institutional guidelines.

### 2.9 *In vivo* experiments in animals

8-12-week-old NOD SCID mice were selected as tumor xenograft model mice, and SW1116 cells were injected into the right side of the buttocks and back of the mice (1×10^6^ cells), 60μl each time to establish a colon cancer xenograft mouse model. On the third day of tumor-bearing, 25 tumor-bearing mice were randomly divided into a blank control group (Untreated), NK92 treatment group, UCB-NK cell treatment group, hESC-NK cell treatment group, and CAR hESC-NK cell treatment group, 5 mice per-group. NK cells were injected into tail vein (1.5×10^7^ cells/100μl), all NK cells were resuspended in 100μl PBS. All the mice were injected once a week for 4 consecutive weeks. In the first week, IL-15 (10ng/mouse) was injected into the tail vein every day; from 1 to 21 days, the interleukin IL-2 (10000U/mouse) was injected into the tail vein every 2–3 days. 50μl of peripheral blood was collected from tumor-bearing mice via tail vein puncture using a disposable sterile blood sampling needle at 7-day intervals. Following erythrocyte lysis (BD Pharm Lyse™ Lysing Buffer, 555899), the harvested leukocytes were stained with Anti-Human CD56 antibody (555518, BD) for flow cytometric analysis. NK cell proportions were calculated as the percentage of human CD56^+^ cells within the total live single-cell gate after exclusion of debris. Prior to tissue collection upon completion of the 42-day observation period, mice were first anesthetized via intraperitoneal injection of sodium pentobarbital at a dosage of 50 mg/kg, followed by humane euthanasia through cervical dislocation. No adverse events were observed throughout the experimental procedures, and all operations were performed to minimize animal distress to the greatest extent possible. All animal operations and experimental protocols were performed in accordance with the approved institutional animal ethical guidelines and complied with international animal welfare standards.

### 2.10 Statistical analysis

All data are expressed as mean ± SD. Differences between groups were analyzed using one way ANOVA, with SPSS 17.0 statistical software. *p* < 0.05 regarded statistically significant. *p < 0.05, **p < 0.01, ***p < 0.001, ns not significant.

## 3 Results

### 3.1 Establishment of CEA-targeted CAR-hESCs with normal hESC characteristics

**Figure 1:**
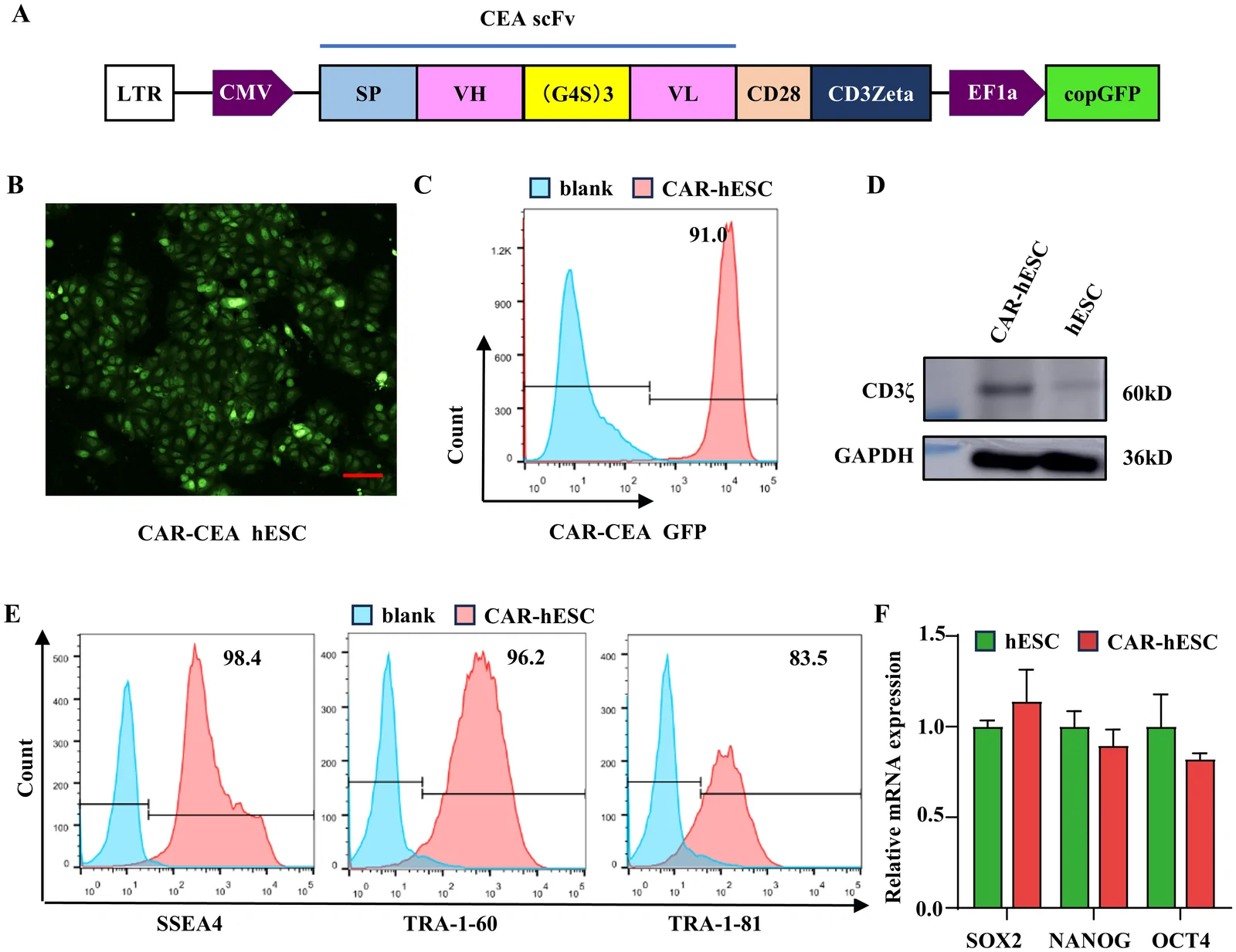
Construction of the CAR-CEA vector and genetic modification of hESCs. **(A)** Schematic diagram of the CAR-CEA expression cassette showing the functional domains; **(B)** Representative fluorescence microscopy image showing GFP expression in CAR-hESCs after lentiviral transduction. (scale bar=100μm); **(C)** Flow cytometric analysis of GFP-positive CAR-hESCs; **(D)** Western blot analysis of CD3ζ expression in CAR-hESCs. GAPDH was used as a loading control; **(E)** Flow cytometric analysis of pluripotency marker expression (SSEA-4, TRA-1-60, and TRA-1-81) in CAR-hESCs; **(F)** qPCR analysis of stemness-related gene expression (SOX2, NANOG, and OCT4) in hESCs before and after CAR modification. Data are presented as mean ± SD from three independent experiments.

To obtain a homogeneous population of CEA-targeted CAR-NK cells, we engineered hESCs to express a CEA-specific CAR via viral infection. A CAR targeting CEA was constructed using the MEF23 scFv, linked to CD28 and CD3ζ signaling domains and a GFP reporter (**Figure 1A**; **Supplementary Figure S1**). hESCs transduced with this construct stably expressed GFP, with 91% of cells being GFP-positive by flow cytometry on undifferentiated CAR-hESCs, prior to the initiation of differentiation (**Figures 1B, C**). Western blot confirmed expression of the CAR-associated CD3ζ chain in these cells (**Figure 1D**). CAR-hESCs maintained pluripotency, as evidenced by high expression of surface markers SSEA4, TRA-1-60, and TRA-1–81 by flow cytometry (**Figure 1E**), and showed no significant difference in mRNA levels of core pluripotency genes (*SOX2, NANOG, OCT4*) compared to wild-type hESCs (**Figure 1F**). These data confirm that the generated CAR-hESC line retains its pluripotent identity.

### 3.2 Hematopoietic stem/progenitor cells differentiation from CAR-hESCs

**Figure 2:**
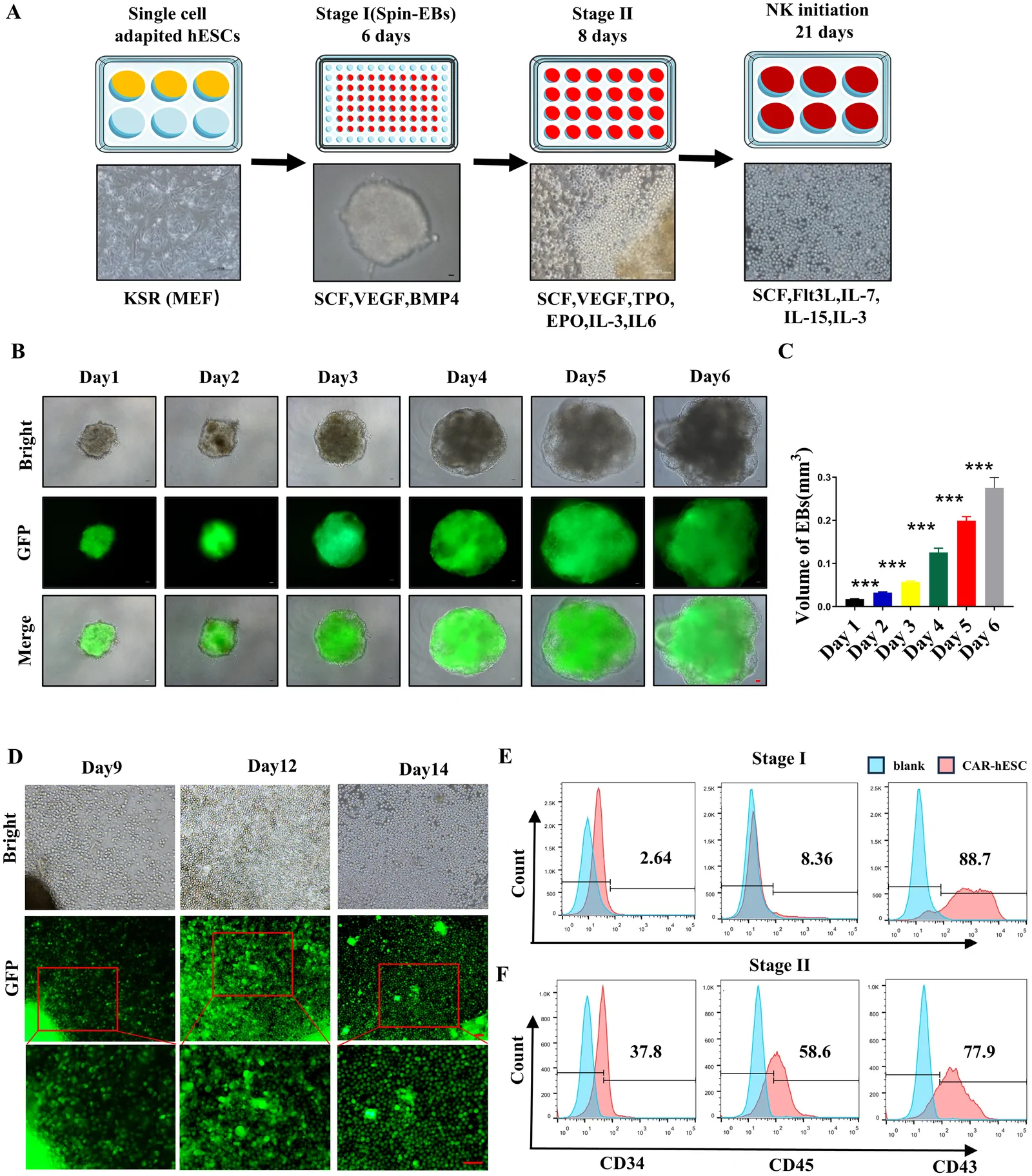
Induction of hematopoietic stem/progenitor cells differentiation from CAR-hESCs. **(A)** Schematic diagram of the induction process from CAR-CEA-hESCs into NK cells; **(B)** Representative images showing morphological changes of EBs during Stage I differentiation. (scale bar=50μm); **(C)** EB volume was calculated from the diameters of EB spheres measured in randomly selected microscopic fields. Asterisks indicate the P values for day-to-day comparisons of EB volume between adjacent time points; **(D)** Representative images depicting the differentiation and maturation of hematopoietic stem/progenitor cells during Stage II (scale bar=50μm); **(E, F)** Flow cytometric analysis of CD34, CD45 and CD43 expression. **(E)** stage I; **(F)** stage II. Data are presented as mean ± SD from three independent experiments. ***p <0.001.

To achieve *in vitro* induction of CEA-targeted CAR-NK cell differentiation and maturation, a three-stage induction method (**Figure 2A**) was used. The first stage was induced by the Spin EB method in 96-well plates to generate EBs. This stage lasted 6 days, during which cytokines SCF, VEGF, and BMP4 were added. As the induction time extends, the Spin EBs gradually increase in size, accompanied by a progressive increase in the number of hematopoietic stem/progenitor cells growing in suspension (**Figures 2B, C**). In the second stage, the EB spheroids were transferred into 24-well plates and cultured with cytokines SCF, VEGF, TPO, EPO, IL-3, and IL-6 for 8 days. This stage promotes the differentiation of hematopoietic stem/progenitor cells into the lymphoid lineage, giving rise to CD34^+^CD45^+^ hematopoietic progenitors cells (**Figures 2D, E**).

### 3.3 Efficient generation of mature CAR ESC-NK cells from CAR-hESCs

**Figure 3:**
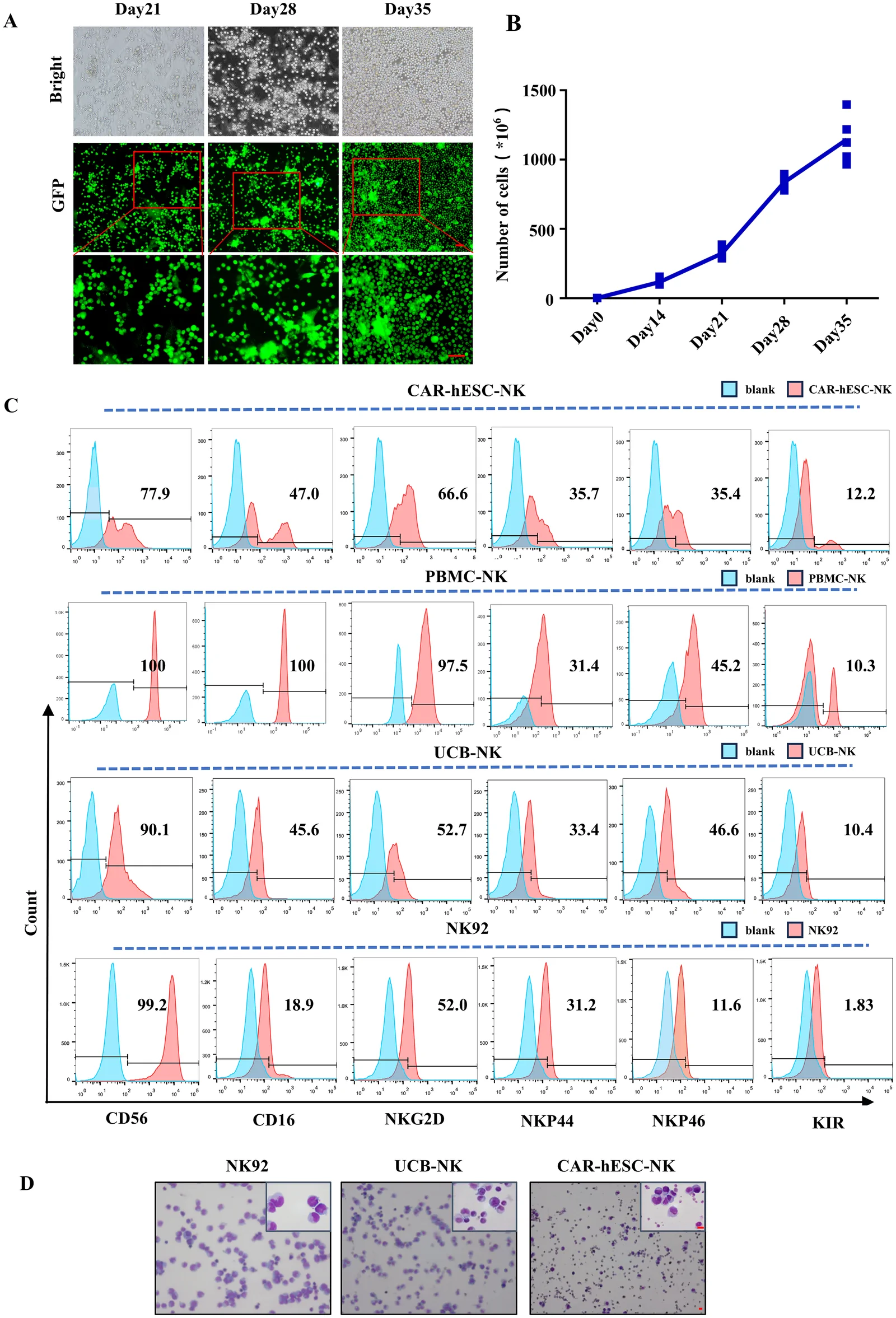
Generation of mature CAR-CEA NK cells. **(A)** Representative images depicting the maturation of CAR-CEA NK cells during Stage III (scale bar=50μm); **(B)** Cell number changes during the differentiation of CAR-CEA-hESCs toward NK cells; **(C)** Flow cytometric analysis of NK cell markers expression from different sources; **(D)** Giemsa staining of NK cells from different sources. (scale bar=50μm).

In the final stage, NK cell induction was performed in 6-well plates. Mature NK cells were obtained following a 3-week exposure to cytokines SCF, Flt3L, IL-7, IL-15, and IL-3 (**Figure 3A**). As the induction time prolonged, the yield of induced cells progressively increased (**Figure 3B**). The expression levels of key molecular markers on NK cells were assessed by flow cytometry. Consistent with widely used and well-characterized NK cell references-including peripheral blood mononuclear cell derived NK cells (PBMC-NK), umbilical cord blood derived NK cells (UCB-NK), and the NK92 cell line, which serve as reliable benchmarks for phenotypic and functional assays, the CAR-CEA NK cells exhibited a CD56^+^CD16^+^ phenotype, along with high surface expression of NKp44, NKp46, and NKG2D. In contrast, KIR expression remained low, indicating normal NK cell functionality (**Figure 3C**; **Supplementary Figure 2**). Furthermore, Giemsa staining demonstrated that the induced NK cells displayed morphological characteristics typical of normal NK cells (**Figure 3D**). These results demonstrated that CAR-hESCs can efficiently differentiate into mature CAR-CEA NK cells.

### 3.4 CAR modification enhances NK cytotoxicity against CEA-positive colorectal cancer cells

**Figure 4:**
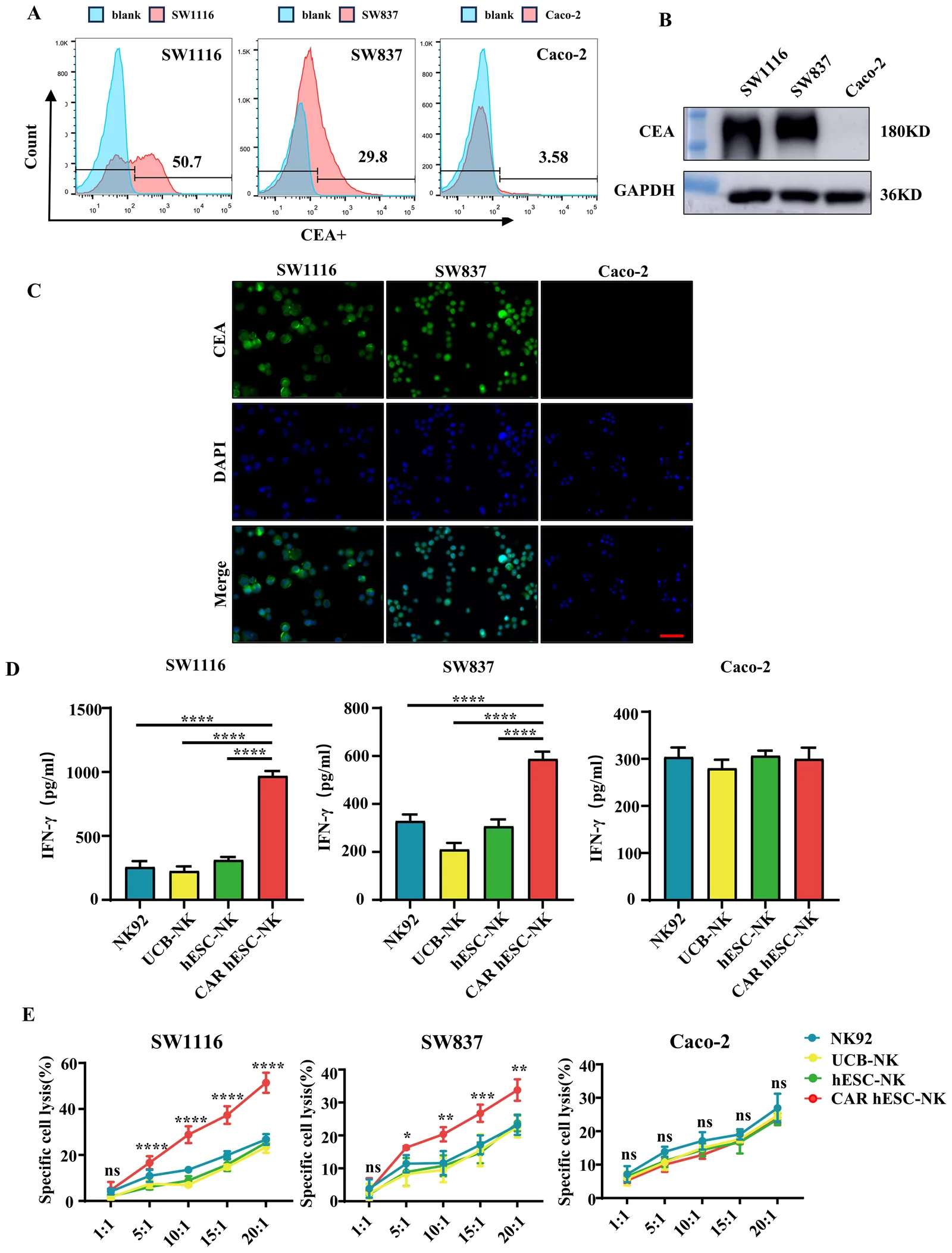
CAR modification enhances NK cytotoxicity against CEA-positive colorectal cancer cells. **(A)** Flow cytometric analysis of CEA expression in colorectal cancer cell lines SW1116, SW837 and Caco-2; **(B)** Western blot analysis of CEA in SW1116, SW837 and Caco-2; **(C)** Representative immunofluorescence staining of CEA in cytospin preparations of SW1116, SW837, and Caco-2. Nuclei were counterstained with DAPI (scale bar=50μm); **(D, E)** IFN-γ **(D)** and LDH **(E)** release assay for assessing the tumor killing ability of NK cells from different sources; Asterisks indicate adjusted P values for comparisons between CAR hESC-NK and hESC-NK at each E:T ratio. Data are presented as mean ± SD from three independent experiments. *p <0.05; **p <0.01; ***p <0.001; ****P < 0.0001; ns, not significant.

For *in vitro* evaluation of CAR hESC-NK cell-mediated cytotoxicity, three colorectal cancer cell lines (SW1116, SW837 and Caco-2) were selected. CEA expression was assessed by flow cytometry, western blotting, and immunofluorescence, revealing high expression in SW1116 and SW837 but not in Caco-2 (**Figures 4A–C**). Furthermore, the tumor-killing function of NK cells was assessed via IFN-γ release and LDH assays. NK92 cells, UCB-NK cells, and hESC-NK cells all exhibited cytotoxic effects against colorectal cancer cell lines, with no significant differences among different NK sources. In contrast, CAR hESC-NK cells exhibited significantly enhanced killing activity specifically against CEA-positive cell lines (SW1116 and SW837 but not Caco-2) compared to hESC-NK cells (**Figures 4D, E**). NK92 cells and UCB-NK cells were additionally included in the assays as reference populations to provide a benchmark for baseline NK cell tumoricidal activity against colorectal cancer targets. These results suggest that CAR modification enhances NK cytotoxicity against CEA-positive colorectal cancer cells.

### 3.5 CAR-hESC NK cells exhibit potent anti-tumor activity against colorectal cancer

**Figure 5:**
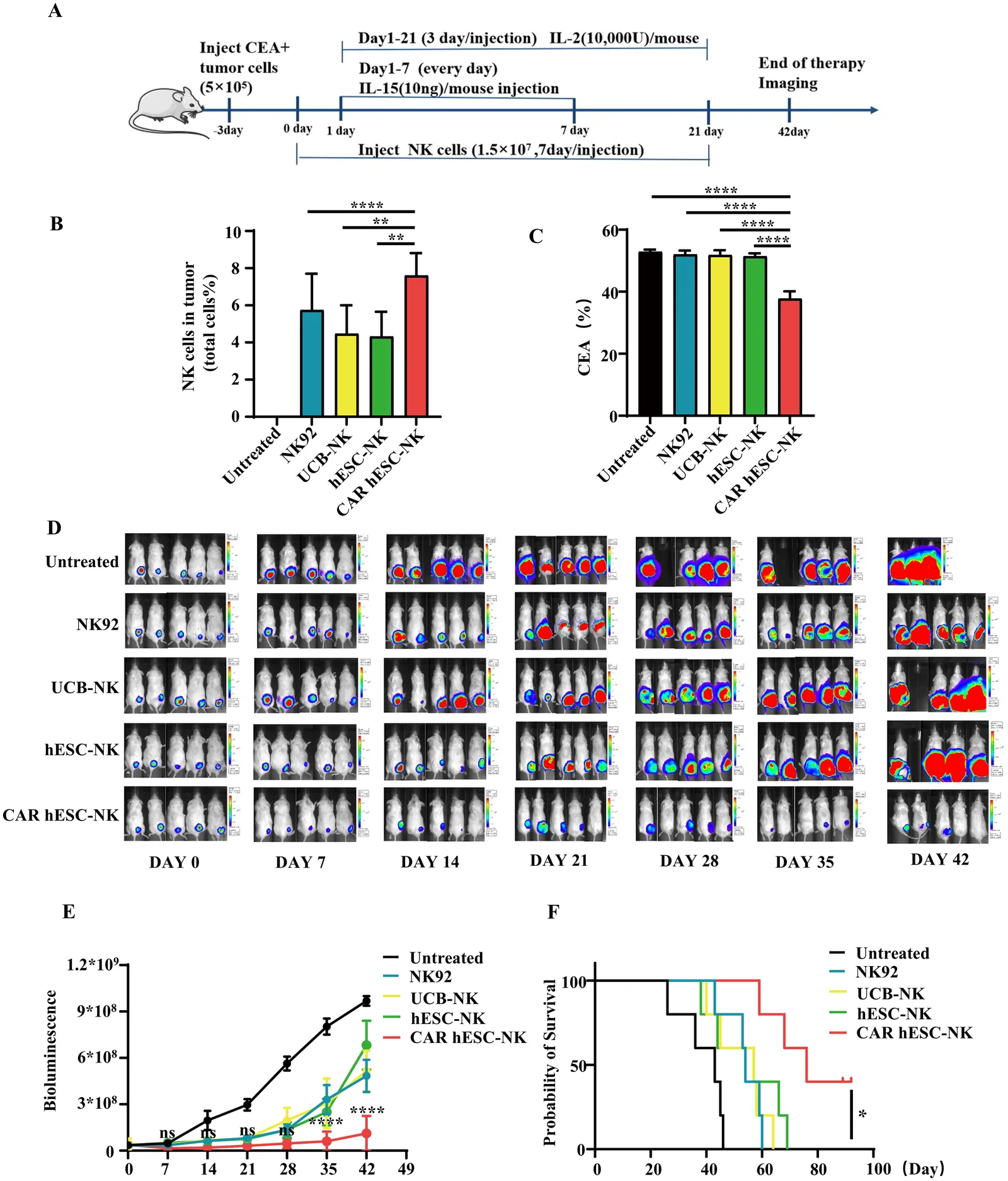
CAR-CEA NK cells exhibit potent anti-tumor activity against colorectal cancer. **(A)** Schematic of the *in vivo* treatment schedule; **(B)** Flow cytometric analysis of NK cell infiltration in tumor tissue after treatment in each group; **(C)** Flow cytometric analysis of CEA expression in tumor tissue after treatment in each group; **(D)** Bioluminescence images showing dynamic tumor growth at indicated time points; **(E)** Quantification of tumor bioluminescence intensity over time. Asterisks indicate adjusted P values for comparisons between CAR hESC-NK and hESC-NK at each time point; **(F)** Survival curve comparing overall survival across treatment groups. Asterisks indicate adjusted P values for comparisons between CAR hESC-NK and hESC-NK at each time point. n=5, * p <0.05; ** p <0.01; *** p <0.001; **** P < 0.0001, ns: not significant.

As outlined in the flowchart presented in **Figure 5A**, functional assessment was conducted at the animal level. SW1116 cells were injected into the right flank and back of the mice to establish a colon cancer xenograft mouse model. The proportion of NK cells infiltrating the tumor tissue was analyzed by flow cytometry, which revealed that the CAR hESC-NK treatment group exhibited a significantly higher degree of NK cell infiltration compared with the other groups (**Figure 5B**). Furthermore, CEA protein expression in colon cancer tissues was evaluated using flow cytometry and immunofluorescence (**Figure 5C**; **Supplementary Figure 3A**). The results demonstrated that the expression of CEA in the CAR hESC-NK-treated group was markedly lower than that observed in the four control groups. Weekly *in vivo* dynamic imaging was conducted to track changes in tumor volume. The results demonstrated that tumors in the NK92 cell, UCB-NK cell, and hESC-NK cell treatment groups all exhibited a tendency toward regression. Notably, in the CAR hESC-NK treatment group, tumor volumes began to decrease from the second week onward, with significant shrinkage observed (**Figure 5D, E**; **Supplementary Figure 3B**). Additionally, survival curve analysis revealed that mice receiving CAR hESC-NK therapy had the longest median survival duration (**Figure 5F**). Collectively, these findings indicate that CAR hESC-NK cells exert a therapeutic effect through the targeted elimination of CEA-positive colon cancer cells.

### 3.6 CAR hESC-NK cells exhibit a favorable biosafety profile with no accumulation or organ toxicity

**Figure 6:**
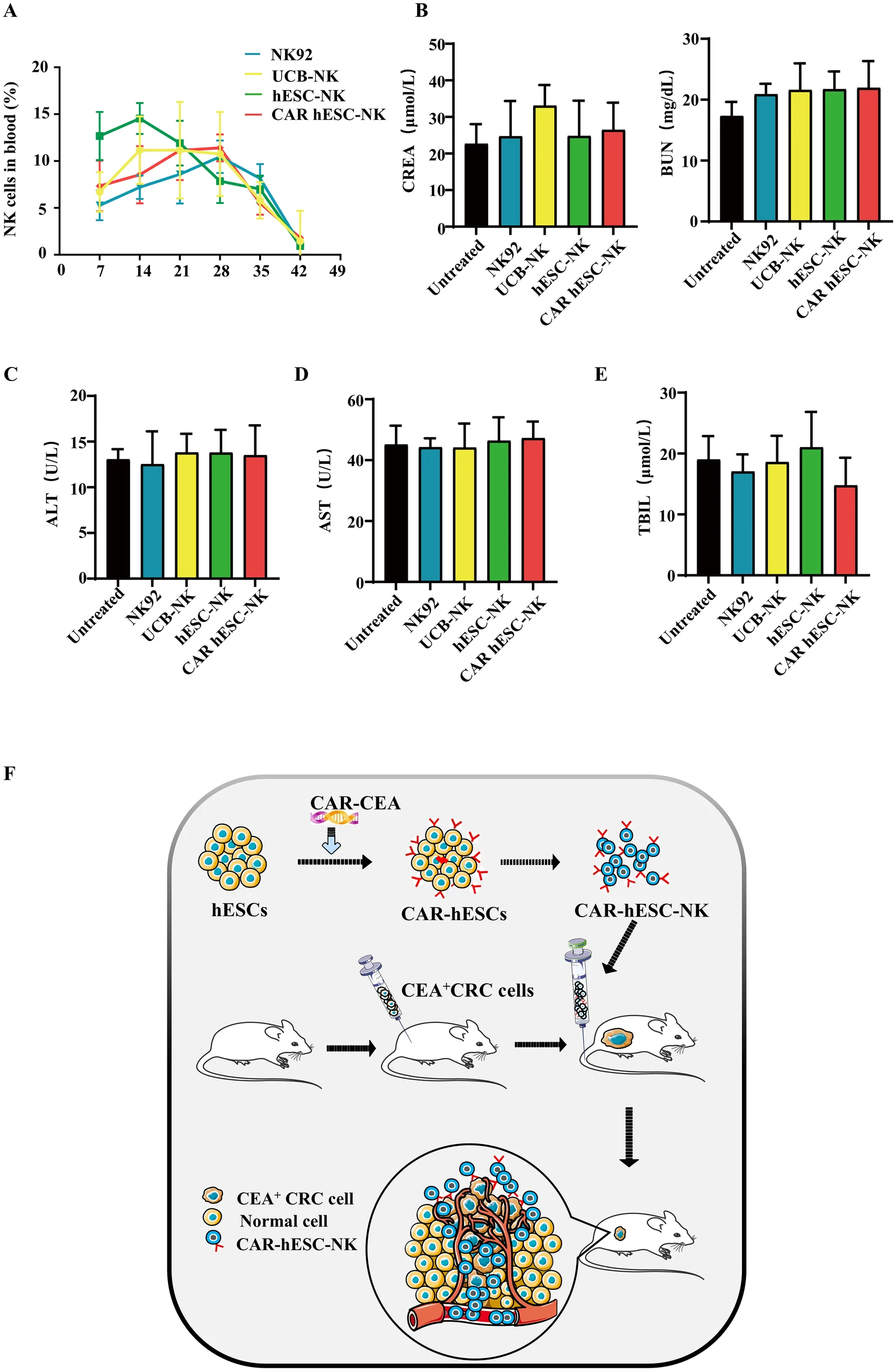
*In vivo* safety profile of CAR-CEA-hESC-NK cell therapy in a colorectal cancer xenograft model. **(A)** Flow cytometry analysis of NK cell proportions in peripheral blood at indicated time points post-treatment; **(B)** Renal function assessed by serum CREA and BUN levels; **(C–E)** Liver function evaluated by serum ALT, AST and TBIL level of mice in each group; **(F)** Model diagram of CEA-Targeted CAR-NK Cells Derived from Embryonic Stem Cells Against Colorectal Cancer.

To evaluate the safety of CAR hESC-NK treatment, we assessed both the biodistribution of CAR hESC-NK cells in peripheral blood, as well as liver and kidney function markers in treated mice. Peripheral blood analysis revealed that the proportion of NK cells from different sources remained at approximately 10% during treatment. By six weeks, peripheral blood NK cell levels approached zero. And no significant differences in peripheral blood NK cell proportions were observed among the NK92, UCB-NK, hESC-NK, and CAR hESC-NK treatment groups at any time point (**Figure 6A**). Furthermore, no obvious abnormalities in liver and kidney function were detected across treatment groups (**Figures 6B–E**). These results demonstrate a limited *in vivo* lifespan without accumulation, coupled with an absence of organ toxicity, thereby supporting a favorable biosafety profile.

## 4 Discussion

This study investigates the therapeutic potential of CAR-modified NK cells derived from hESCs targeting CEA, a well-established tumor marker in CRC (16). Relative to conventional chemotherapy and targeted agents (e.g., anti-EGFR or anti-VEGF antibodies), our approach offers the advantage of antigen-specific tumor recognition, which may spare normal tissues and reduce systemic adverse effects (32, 33). When compared with autologous CAR-T cells targeting CEA, the hESC-derived CAR-NK platform presents several theoretical advantages in safety and logistics (34, 35). NK cells are intrinsically less prone to trigger severe cytokine release syndrome and neurotoxicity, and they do not cause GvHD, making them suitable for allogeneic use. Moreover, in contrast to CAR-NK92 cells, which require irradiation prior to infusion due to their tumor origin, the hESC-derived NK cells generated in this study constitute a renewable, highly scalable, and standardized “off-the-shelf” product (20, 21). This platform effectively circumvents the challenges of donor variability and the complex manufacturing processes typically encountered with primary NK cells (36). The specific CEA-CAR structure employed herein, designed for optimal NK cell activation, mediates potent and specific cytotoxicity against CEA-positive colorectal cancer cells, an outcome consistent with the efficacy observed in other CEA-targeted immunotherapies (37, 38). Collectively, these findings support the growing consensus that CAR-NK cells represent a promising alternative for solid tumors, particularly in their capacity to overcome the immunosuppressive TME.

The three-stage differentiation protocol utilized in this study has proven pivotal in generating a stable population of CAR hESC-NK cells with robust anti-tumor activity. This systematic approach not only ensures the maintenance of pluripotency markers throughout the differentiation process but also promotes the maturation of NK cells exhibiting enhanced proliferation and functionality. Such advancements could potentially lead to improved NK cell therapies that harness the full therapeutic potential of these immune effectors in treating malignancies. Furthermore, the ability to produce a uniform and potent NK cell population from hESCs may also address the limitations posed by traditional sources of NK cells, thus enhancing the accessibility and feasibility of NK cell-based therapies in clinical settings. Moreover, the immune mechanisms elucidated in this study highlight the importance of NK cell specificity in the context of colorectal cancer treatment. Recent early-phase trials have demonstrated encouraging safety and preliminary antitumor activity of CAR-NK cells targeting various antigens in advanced CRC, particularly in patients refractory to standard therapies (39, 40). The demonstrated cytotoxicity of CAR hESC-NK cells against CEA-positive colorectal cancer cell lines, coupled with their favorable safety profile, underscores the potential of these engineered cells to induce robust anti-tumor responses.

Notably, the MEF23 scFv incorporated into the CAR construct exhibits no cross-reactivity with members of the murine CEACAM family. Given the absence of endogenous human CEA expression in the normal tissues of xenograft tumor-bearing mice, this *in vivo* model cannot fully recapitulate clinically relevant on-target, off-tumor effects associated with CEA-targeted cellular immunotherapy. Future investigations employing human CEA-expressing transgenic mouse models are warranted to rigorously evaluate on-target, off-tumor effects and advance the translational safety assessment of this therapeutic strategy. While GFP fluorescence served as a reliable surrogate for tracking transgene expression in this study, we acknowledge that it provides an indirect readout of CAR expression. Although the correlation between GFP and CAR transcription is generally reliable, potential discrepancies at the protein level remain. Consequently, our conclusions based on GFP tracing should be viewed with this caveat in mind, and direct quantification of surface CAR protein should be prioritized in future studies to unambiguously verify CAR persistence.

With respect to resistance, tumors may evade CEA-directed CAR-NK cells through antigen loss or downregulation under selective pressure, a phenomenon well documented in CAR-T trials (15, 41). Additional resistance could arise from the highly immunosuppressive colorectal tumor microenvironment, enriched with TGF-β, adenosine, and myeloid-derived suppressor cells, which are known to dampen NK cell cytotoxicity. Rational combination approaches, such as co-administration with TGF-β inhibitors, immune checkpoint blockers, or agents that remodel the extracellular matrix, could potentially circumvent these obstacles and represent an important direction for further investigation This specificity is particularly crucial given the challenges posed by the TME, which often impairs the efficacy of conventional immunotherapies. The findings suggest that modulation of specific immune pathways may enhance the anti-tumor activity of NK cells, thereby providing a foundation for the development of novel immunotherapeutic interventions that can effectively combat colorectal cancer and other malignancies characterized by similar antigenic profiles (42). This study not only contributes to the growing body of literature on CAR-NK cell therapies but also emphasizes the need for further research to optimize these approaches for clinical application.

Building upon the presented preclinical evidence, key avenues require further investigation for clinical translation. First, despite the current CAR architecture’s robust anti-tumor activity, future studies should incorporate functional elements to enhance persistence and overcome the immunosuppressive TME (43). The inclusion of cytokine armoring, such as membrane-bound IL-15, may promote CAR-NK cell survival and expansion following adoptive transfer without exogenous cytokine support (44),while co-expressing chemokine receptors targeting colorectal cancer-upregulated ligands could improve tumor infiltration and metastatic disease efficacy (45, 46). Furthermore, batch-to-batch consistency is critical for hESC-derived CAR-NK cells as off-the-shelf products (47). Though a well-characterized master cell bank minimizes starting material variability, rigorous validation of differentiation parameters is essential for process robustness (48). Comprehensive release criteria must guarantee each lot meets potency and safety standards (49). Standardized, scalable GMP-compliant production coupled with quality control systems positions the hESC-derived CAR-NK platform to deliver consistent, readily available clinical immunotherapeutics.

## 5 Conclusions

In summary, this study successfully establishes a renewable, scalable source of off-the-shelf CAR-NK cells by engineering hESCs to express a CEA-specific chimeric antigen receptor. The resulting CAR hESC-NK cells exhibit potent and selective cytotoxicity against CEA-high colorectal cancer cells, robust cytokine release, and significant *in vivo* tumor suppression without detectable off-target toxicity (**Figure 6F**). By providing a stable and homogeneous cell product, this platform overcomes key limitations of current immunotherapies, including limited efficacy and cell source availability. Our findings support CAR hESC-NK cells as a promising, ready-to-use therapeutic strategy not only for CEA-positive colorectal cancer but also for other CEA-expressing malignancies, warranting further clinical translation.

## Data Availability

The original contributions presented in the study are included in the article/**Supplementary Material**. Further inquiries can be directed to the corresponding authors.
