# Supplementary material for: CEA-targeted CAR-NK cells derived from embryonic stem cells exhibit potent anti-tumor activity against colorectal cancer
Source: Front Immunol. 2026 Aug 28;17:1901058. doi: 10.3389/fimmu.2026.1901058 (PMC13561994; doi:10.3389/fimmu.2026.1901058)
Supplement: Supplementary file 1 [file DataSheet1.pdf]

Supplementary Material

1 Supplementary Figures

A

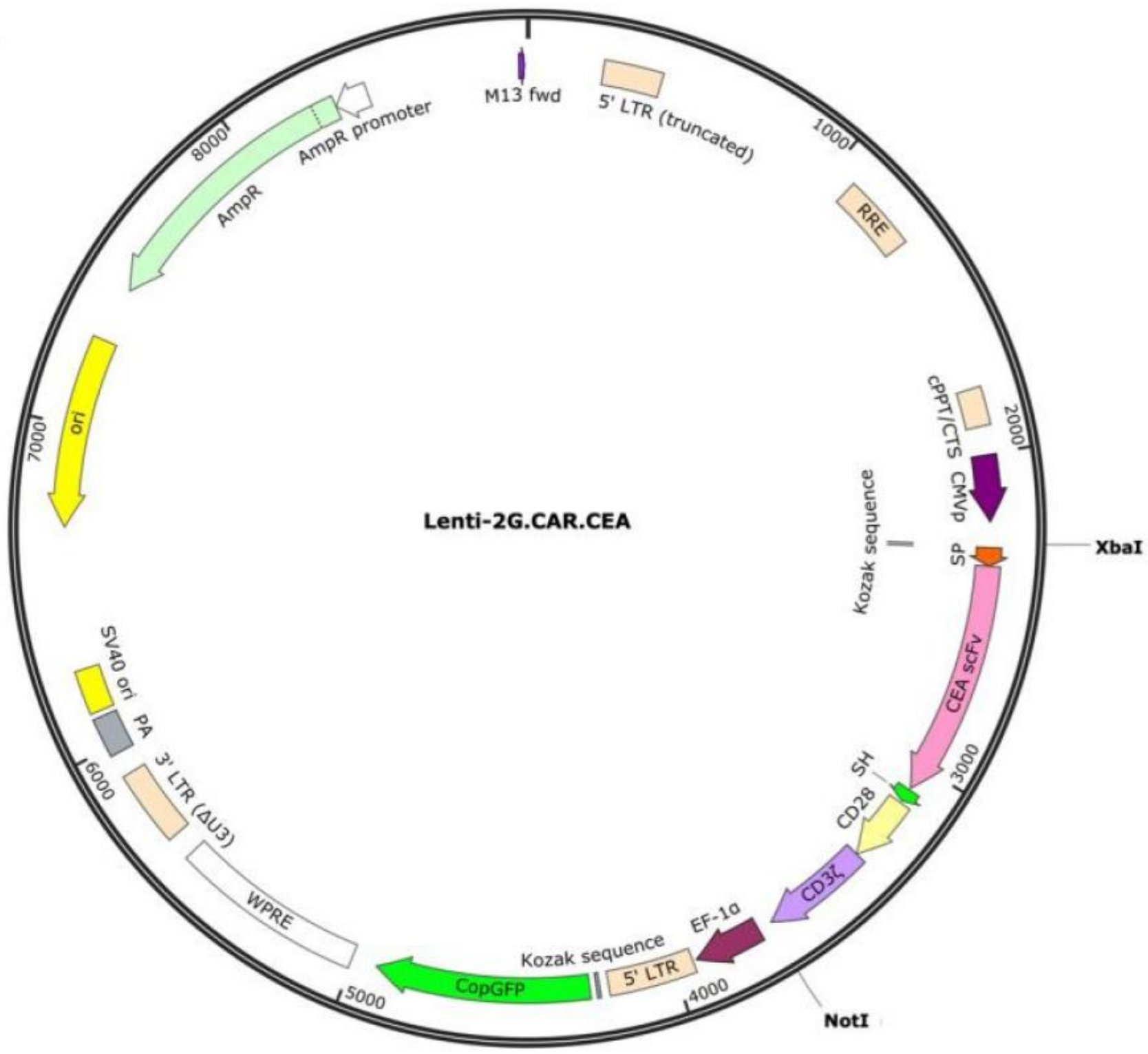

Figure S1. (A) Plasmid map of the lentiviral vector carrying the CEA-CAR expression cassette.

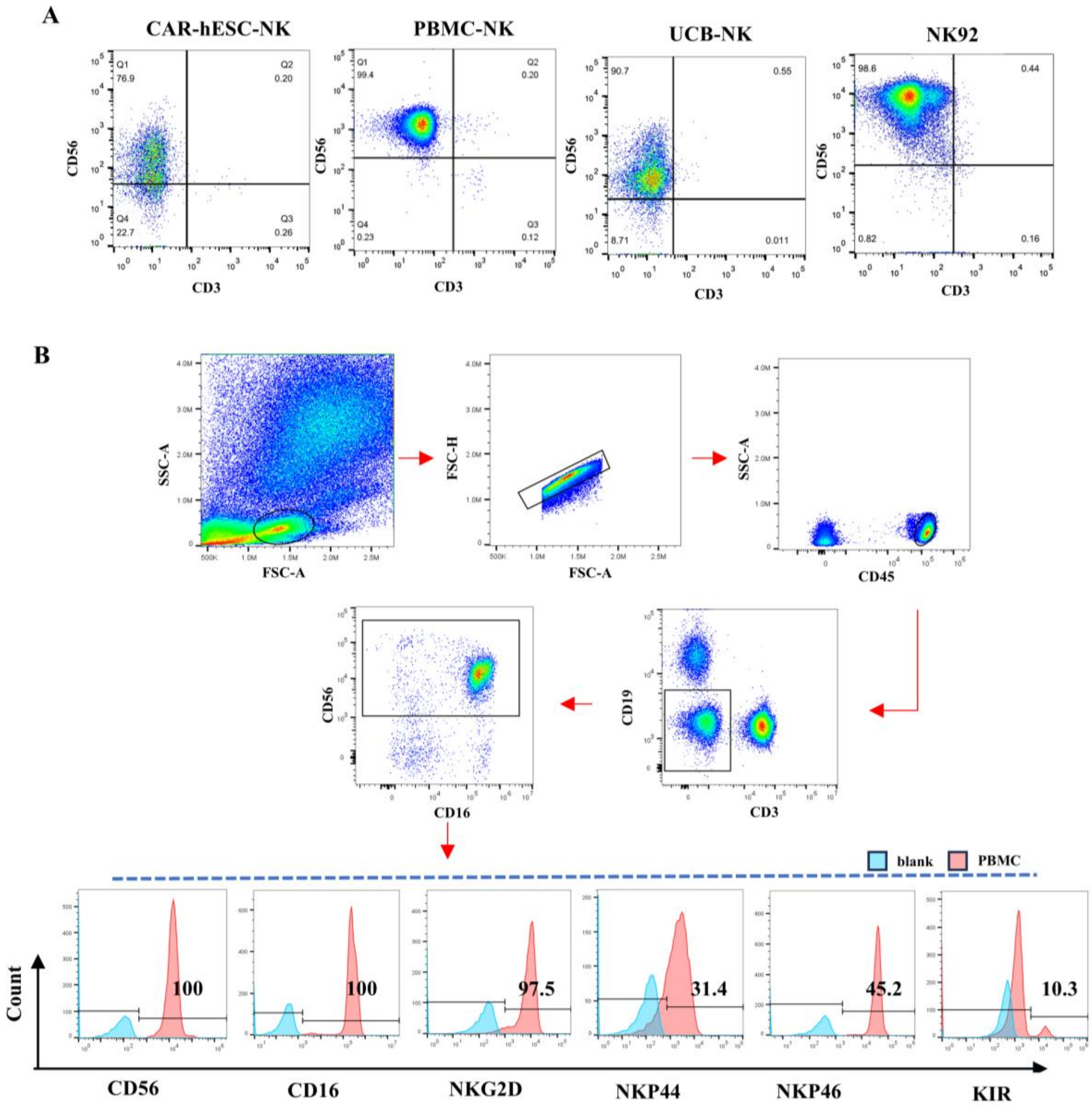

Figure S2. **(A)** Flow cytometric analysis of NK cell markers expression from different sources; **(B)** PBMC were isolated from the peripheral blood of healthy adult donors, and the expression profile of surface markers was characterized via a flow cytometric gating strategy.

A

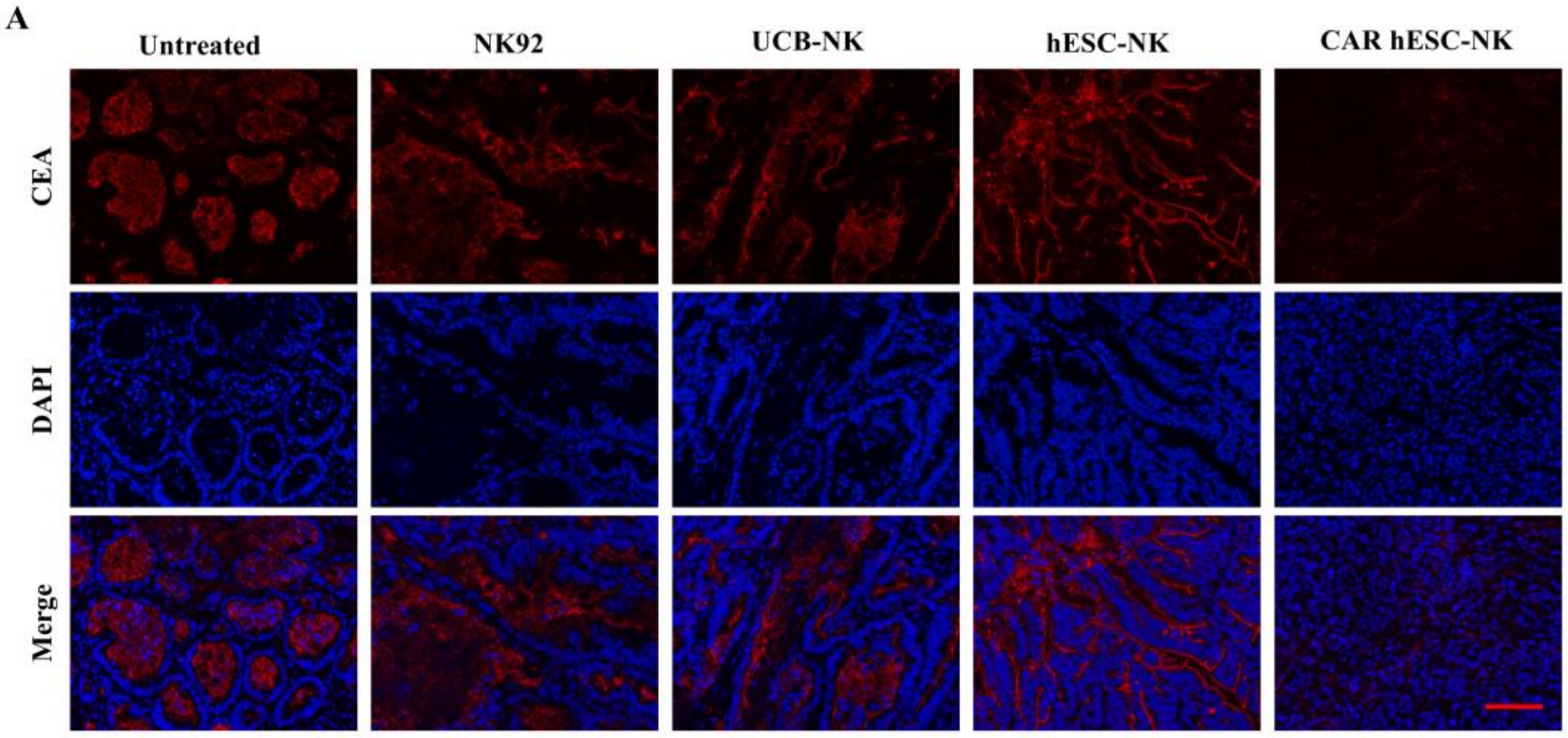

B

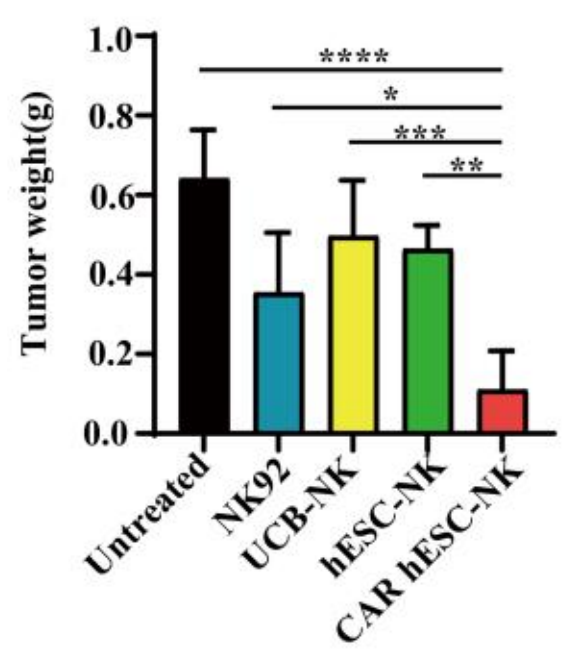

Figure S3. (A) Immunofluorescence detection of CEA expression in tumor tissue after treatment in each group. (scale bar=100μm); **(B)** Histogram of tumor weight in different treatment groups. * p <0.05; ** p <0.01; *** p <0.001; **** p <0.0001

Figure 1

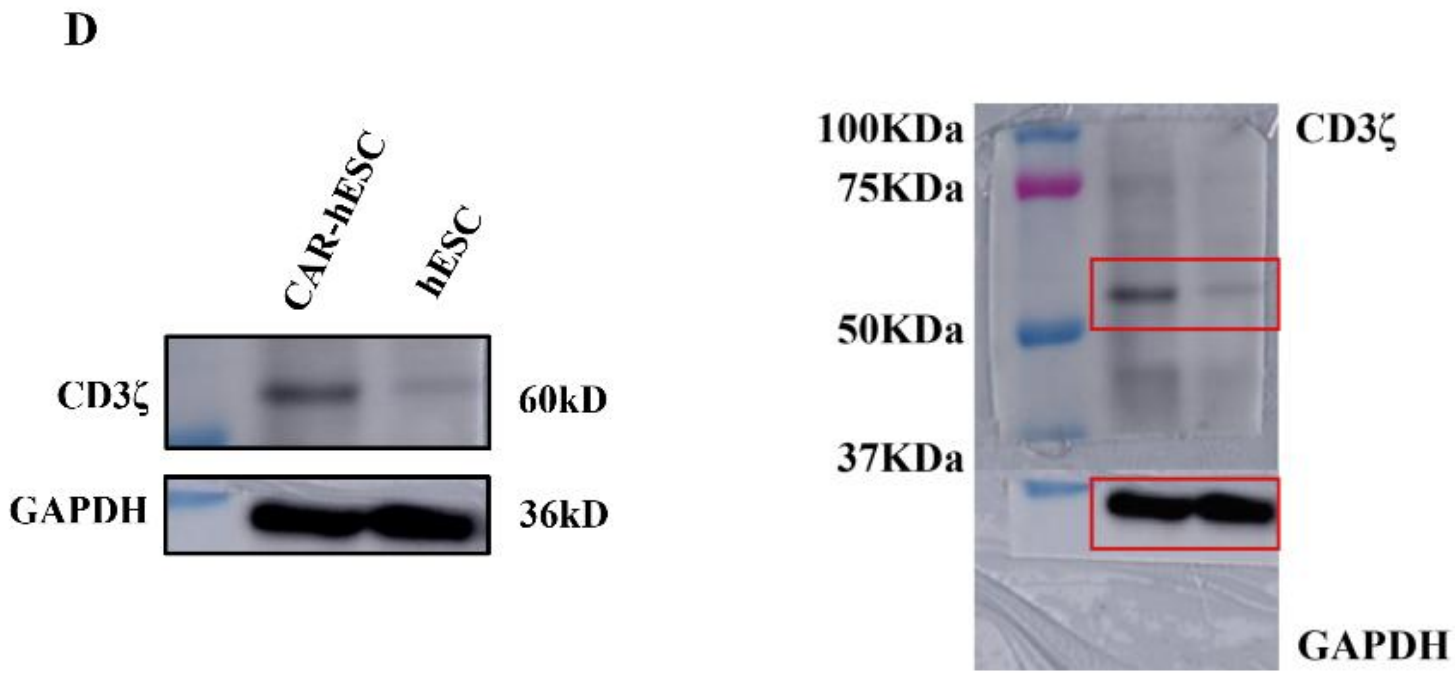

Figure 4

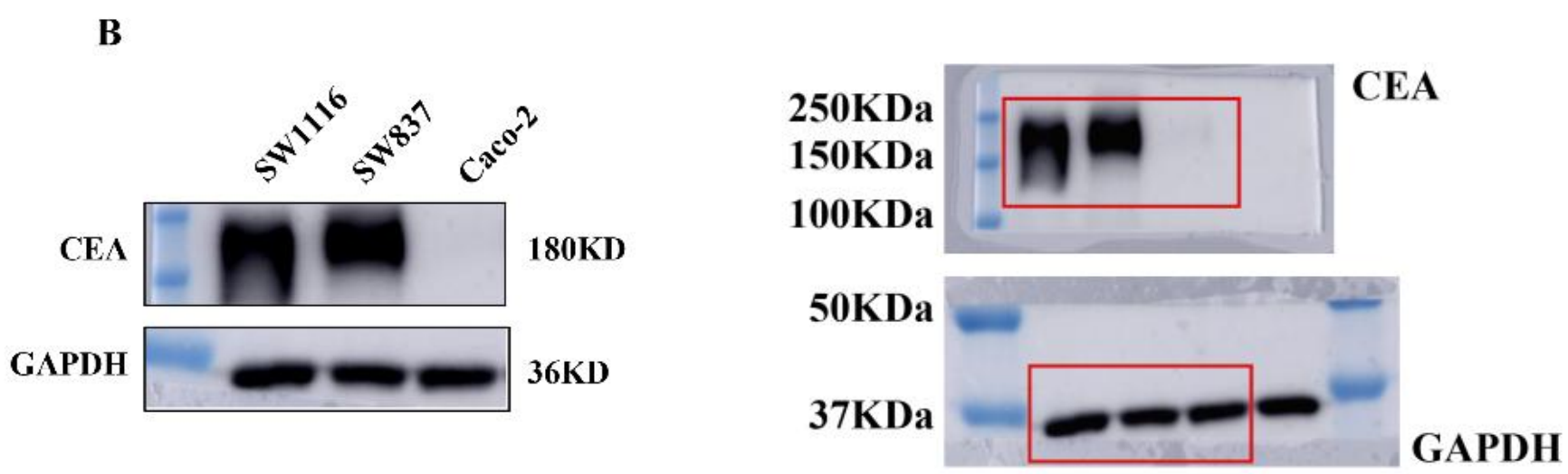

Figure S4. The original, uncropped Western blot images of Figure 1D and Figure 4B.
